# Impact of Natural Blind Spot Location on Perimetry

**DOI:** 10.1038/s41598-017-06580-7

**Published:** 2017-07-21

**Authors:** Mengyu Wang, Lucy Q. Shen, Michael V. Boland, Sarah R. Wellik, Carlos Gustavo De Moraes, Jonathan S. Myers, Peter J. Bex, Tobias Elze

**Affiliations:** 1 Schepens Eye Research Institute, Harvard Medical School, Boston, MA USA; 2 Mass. Eye and Ear Infirmary, Harvard Medical School, Boston, MA USA; 3 Wilmer Eye Institute, Johns Hopkins University School of Medicine, Baltimore, MD USA; 4 Bascom Palmer Eye Institute, University of Miami School of Medicine, Miami, FL USA; 5 Edward S. Harkness Eye Institute, Columbia University Medical Center, New York, NY USA; 6 Wills Eye Hospital, Thomas Jefferson University, Philadelphia, PA USA; 7 Department of Psychology, Northeastern University, Boston, MA USA

## Abstract

We study the spatial distribution of natural blind spot location (NBSL) and its impact on perimetry. Pattern deviation (PD) values of 11,449 reliable visual fields (VFs) that are defined as clinically unaffected based on summary indices were extracted from 11,449 glaucoma patients. We modeled NBSL distribution using a two-dimensional non-linear regression approach and correlated NBSL with spherical equivalent (SE). Additionally, we compared PD values of groups with longer and shorter distances than median, and larger and smaller angles than median between NBSL and fixation. Mean and standard deviation of horizontal and vertical NBSL were 14.33° ± 1.37° and −2.06° ± 1.27°, respectively. SE decreased with increasing NBSL (correlation: r = −0.14, p < 0.001). For NBSL distances longer than median distance (14.32°), average PD values decreased in the upper central (average difference for significant points (ADSP): −0.18 dB) and increased in the lower nasal VF region (ADSP: 0.14 dB). For angles in the direction of upper hemifield relative to the median angle (−8.13°), PD values decreased in lower nasal (ADSP: −0.11 dB) and increased in upper temporal VF areas (ADSP: 0.19 dB). In conclusion, we demonstrate that NBSL has a systematic effect on the spatial distribution of VF sensitivity.

## Introduction

The natural blind spot location (NBSL) represents an absolute scotoma in the visual field (VF) induced by the area of the optic disc on the retina which lacks of light-detecting photoreceptor cells^1, 2^. NBSL is routinely measured in standard VF testing in order to accurately interpret the VF test results, particularly for the diagnosis of glaucoma. Glaucoma, the second leading cause of irreversible blindness^3, 4^, is a group of ocular diseases of optic neuropathy characterized by optic disc excavation for which elevated intraocular pressure is a major risk factor. Functionally, patients with glaucoma develop loss of VF sensitivity over space, known as visual field loss^5, 6^. In typical clinical glaucoma testing, standard automated perimetry performed by perimeters like the Humphrey Field Analyzer (HFA) (Carl Zeiss Meditec, Dublin, CA) measures the VF by testing the patient response to white light stimuli with different luminance intensities presented at fixed locations in the field of vision on a white background. The pattern of *functional* VF loss caused by glaucoma is weakly associated with the *structural* damage of retinal nerve fiber layer (RNFL) bundles which are formed by the axons of retinal ganglion cells^7–9^. In glaucoma diagnostics, the evaluation of both *functional* VF loss and *structural* damage of RNFL is usually combined to assess glaucoma severity^10, 11^. More specifically, accurate normative databases of VF and RNFL from healthy individuals are critical to precisely diagnose glaucoma in a patient^12, 13^.

Current normative databases of VF and RNFL thickness rely on age and relative spatial location. Presently, RNFL thickness measurements are not quantitatively adjusted for the influence of ocular anatomical factors, although there is large inter-individual variability of RNFL bundles trajectories^14, 15^. Generally, tracing RNFL bundles is difficult due to poor visibility in retinal images^16, 17^. Moreover, there is no clear way to integrate the information of numerous RNFL bundle trajectories into the establishment of a normative RNFL bundle database to be used to assist glaucoma diagnosis. It has been shown that the optic disc location relative to fovea is related to RNFL geometry and should be considered for RNFL profile norms^14, 15, 18^. The diagnosis of glaucoma might be potentially improved by including the variability of the optic disc position relative to fovea. The functional equivalent of the optic disc location, the NBSL is routinely measured in standard with automated perimetry devices as part of the VF measurement. Here, we study how NBSL relative to fixation, the functional counterpart of the optic disc position relative to fovea, affects the spatial distribution of VF sensitivity.

In this work, we quantify the effect of NBSL relative to fixation on the spatial distribution of pattern deviation (PD) values in clinically unaffected VFs. PD values represent VF sensitivity relative to the general height of vision (see Methods section) and are important diagnostic parameters for glaucoma.

## Results

11,449 eyes of 11,449 patients met our selection criteria and were included in this study. The mean ± standard deviation of age and spherical equivalent of refractive error of all patients are 58.5 ± 14.5 years and −0.17 ± 2.47 Diopters, respectively. Note that all VFs with a reported NBSL of (15°, −1°) were excluded for our data analysis due to that the HFA returns a “default” NBSL value of (15°, −1°) in absence of a test result^19^.

**Figure 1:**
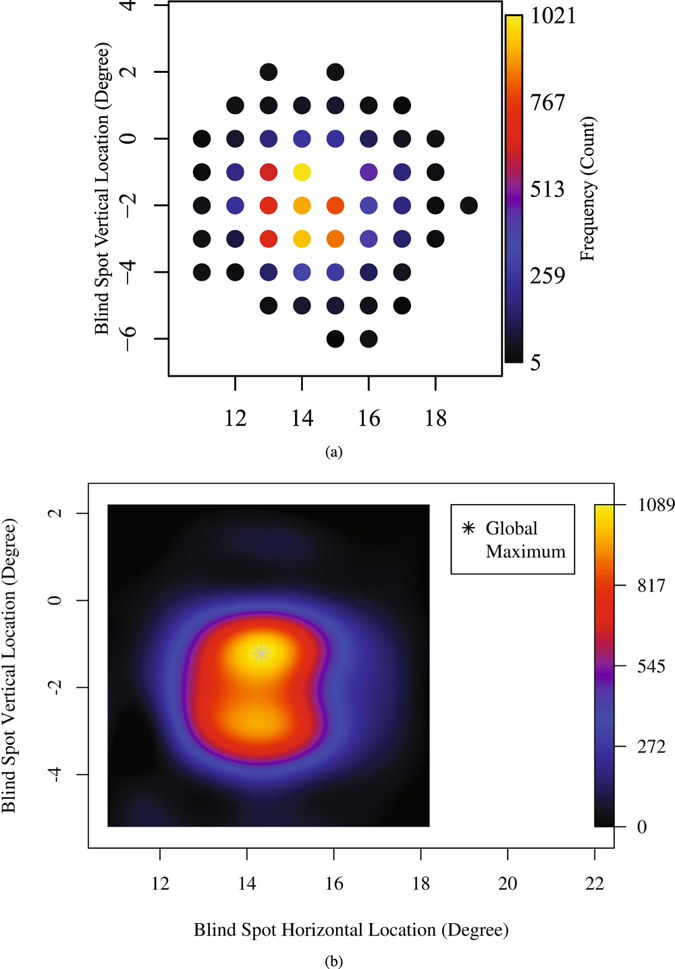
Blind spot distribution over location (**a**) discrete distribution of number of eyes measured at each NBSL and (**b**) continuous distribution of number of eyes at each NBSL calculated by Gaussian process.

Figure 1(a) shows the NBSL distribution over all measured locations. Table 1 shows the detailed frequency distribution of NBSL including NBSLs with less than 5 eyes enclosed by brackets, which were excluded from following Gaussian process regression due to potential reliability issues.

**Table 1:** The detailed spatial distribution of NBSL including NBSLs with less than 5 eyes enclosed by brackets, which were excluded from Gaussian process regression due to possible reliability issues.

Horizontal	7	10	11	12	13	14	15	16	17	18	19	20	21
Vertical													
3	0	0	0	0	0	0	[1]	0	0	0	0	0	0
2	0	0	0	0	7	[3]	6	[2]	[2]	0	0	0	0
1	0	0	[1]	7	47	61	78	34	13	[2]	[1]	0	0
0	0	0	13	74	183	259	243	124	58	8	[3]	0	[1]
−1	0	[1]	21	196	625	1021	NA	447	178	31	[4]	[1]	0
−2	[1]	[2]	45	241	703	909	797	376	186	24	5	0	0
−3	0	0	5	103	667	938	843	415	154	25	[3]	0	0
−4	0	[1]	5	25	150	321	274	125	60	[3]	[2]	0	0
−5	0	0	[1]	[2]	31	75	82	51	15	[2]	[1]	0	0
−6	0	0	0	0	0	[4]	10	8	[3]	0	0	0	0

The NBSL at (15°, −1°) is denoted as missing value since all NBSL of (15°, −1°) is excluded due to the reason we described in Method section.

Figure 1(b) shows the continuous distribution of NBSL computed by the Gaussian process with optimal *σ*_*f*_ = 1.2 and $\overrightarrow{\gamma }=\mathrm{(0.9},\mathrm{0.8)}$ selected based on Bayesian analysis. The NBSL frequency at (15°, −1°) estimated by Gaussian process was 920, which is smaller than the observed maximum of NBSL frequency 1021 at (14°, −1°). The global maximum of the estimated NBSL frequency was 1209 and the location of the global maximum was (14.3°, −1.2°).

**Figure 2:**
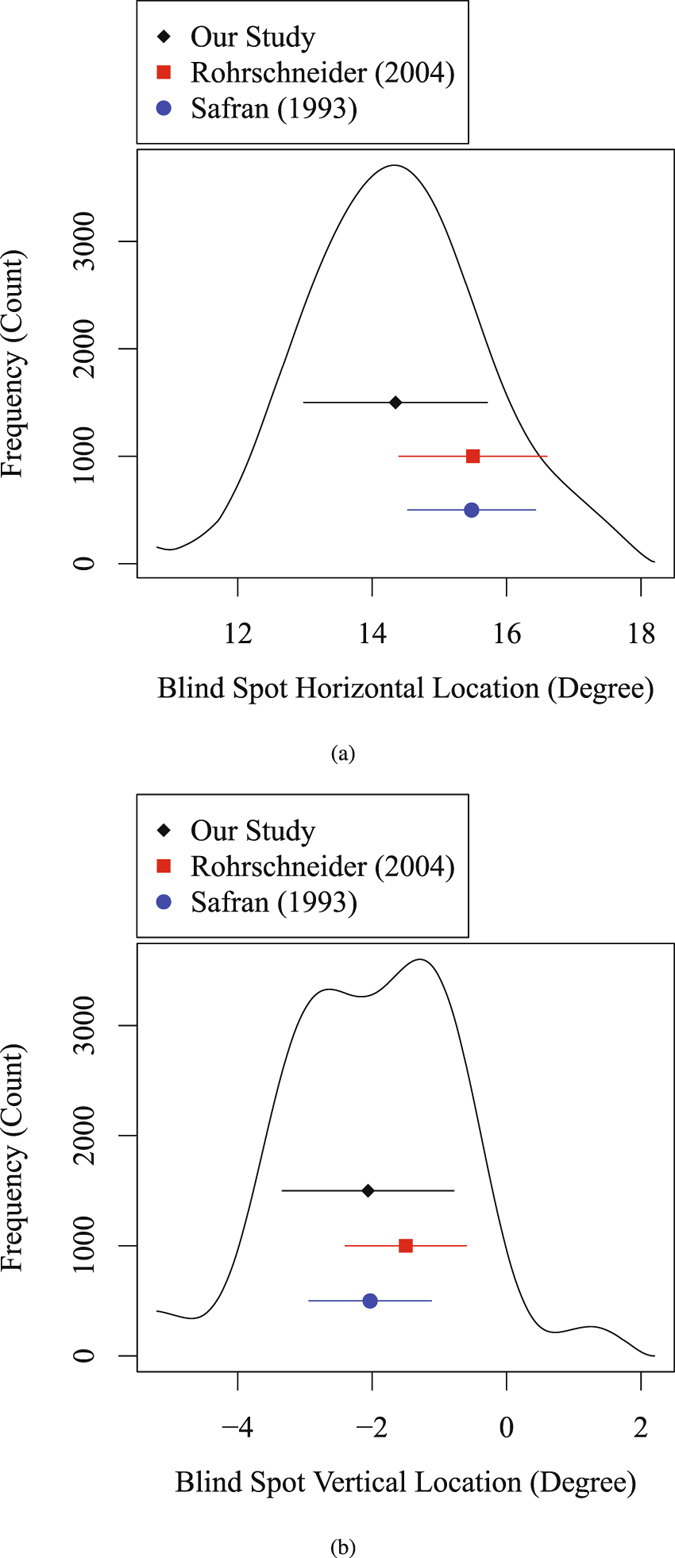
Blind spot marginal distribution in (**a**) horizontal direction and (**b**) vertical direction from the current study and previous studies in literature. Black diamond and line denote the mean and standard deviation of NBSL calculated in our work, blue dot and line denote the mean and standard deviation of NBSL in the work by Safran *et al*.^20^, and red square and line denote the mean and standard deviation of the optic disc location relative to fovea in the work by Rohrschneider^21^.

Figure 2(a) and (b) show the horizontal and vertical marginal distribution of the NBSL frequency in comparisons to the documented mean and standard deviation of NBSL^20^ and optic disc location^21^ in the literature. Note that in the work by Rohrschneider, the optic disc locations relative to fovea were measured instead of NBSL. The average horizontal NBSL (14.35° ± 1.36°) from our study was significantly (*p* < 0.001) different from the measured horizontal location (15.50° ± 1.10°) of the optic disc (the physiological equivalent of NBSL) in the study by Rohrschneider and the measured horizontal NBSL (15.48° ± 0.95°) in the study by Safran *et al*. The average vertical NBSL (−2.06° ± 1.28°) from our study was significantly (*p* < 0.001) different from the measured vertical location (−1.50° ± 0.90°) of the optic disc (the physiological equivalent of NBSL) in the study by Rohrschneider, and not significantly different from the measured vertical NBSL (−2.03° ± 0.91°) in the study by Safran *et al*.

**Figure 3:**
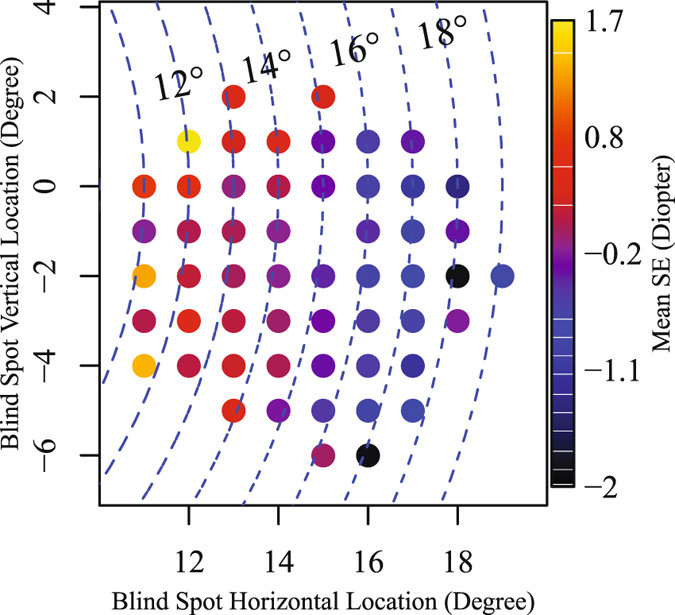
Average values of spherical equivalent over eyes at each NBSL. Blue dashed arc denotes the NBSL distances from fixation.

Figure 3 shows the average value of spherical equivalent (SE) over all eyes measured at each NBSL. Note that since the NBSL measurements were missing at the location (15°, −1°), average of SE was not calculated at (15°, −1°). We observed a negative correlation (r = −0.14, p < 0.001) between NBSL distance from fixation and spherical equivalent of refractive error. As the patient became more myopic (negative refractive error), the distance between NBSL and fixation increased and vice versa.

**Figure 4:**
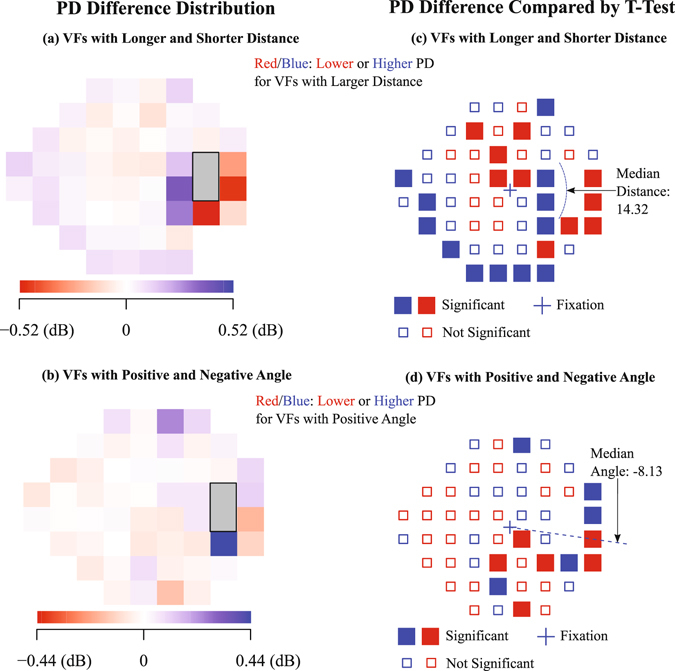
PD value difference effects with respect to NBSL relative to fixation. PD difference compared by t-test (**a**) and spatial distribution (**b**) between the group with NBSL distances longer or equal than the median of all NBSL distances and the group with NBSL distances shorter than the median of all NBSL distances. PD difference compared by t-test (**c**) and spatial distribution (**d**) between the group with NBSL angles larger or equal than the median of all NBSL angles and the group with NBSL angles smaller than the median of all NBSL angles. P-values less than 0.05 after multiple comparison correction with false discovery rate were considered as significant.

Figure 4(a) and (b) show the PD value difference distribution with respect to NBSL relative to fixation and illustrates the effect of distance from fixation and angle relative to fixation. The median NBSL distance and angle were 14.32° and 8.13°, respectively. The maximum absolute differences of average PD values between the two groups divided by median distance and angle are 0.52 dB and 0.44 dB, respectively. The distance effect of NBSL was more noticeable around the default NBSL (15°, −1°) than other locations, while the angle effect of NBSL was relatively more spatially distributed over the VF. Figure 4(c) and (d) show the PD value difference effects compared by t-test with significance annotation with respect to NBSL relative to fixation and illustrates the effect of distance from fixation and angle relative to fixation. Figure 4(c) shows the average PD values of the group with NBSL distances longer than median distance decreased (red) in the upper central (average difference for significant points (ADSP): −0.18 dB) and increased (blue) in the lower nasal VF region (ADSP: 0.14 dB). Figure 4(d) shows the average PD values of the group with NBSL angles larger than median angle (more positive) decreased in lower nasal (ADSP: −0.11 dB) and increased in upper temporal VF areas (ADSP: 0.19 dB).

**Figure 5:**
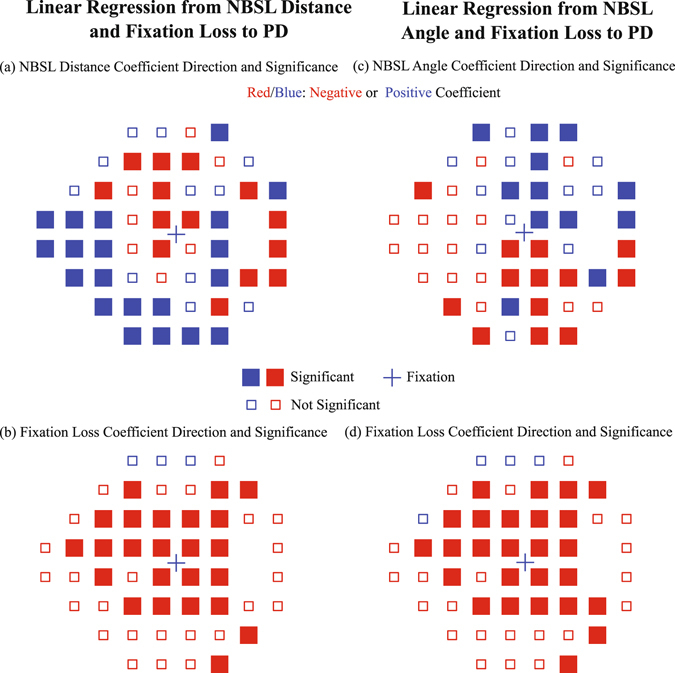
Multivariate linear regression from NBSL distance/angle and fixation loss rate to PD values: (**a**) the NBSL distance coefficient sign and (**b**) the fixation loss coefficient sign for each VF testing locations from the linear regression with NBSL distance and fixation loss rate; (**c**) the NBSL angle coefficient sign and (**d**) the fixation loss coefficient sign for each VF testing locations from the linear regression with NBSL angle and fixation loss rate. P-values less than 0.05 after multiple comparison correction with false discovery rate were considered as significant.

We additionally performed multiple linear regression analyses to disentangle the effects of NBSL distance/angle from possible effects of fixation losses. Figure 5 shows the direction of the coefficients (positive or negative), denoted by blue/red color, respectively, together with the significance of the respective coefficient for each location for (a) NBSL distance, (c) NBSL angle, and the respective coefficients for fixation loss ((b) and (d)). The PD values of all significant locations were negatively associated with fixation rate loss as shown in Fig. 5(b) and (d). Those significant locations that were negatively associated with PD values spread over most regions of the VF.

## Discussion

In this study, we provide an empirical distribution of blind spot centers measured by a standard perimeter for glaucomatous visual field testing. To our best knowledge, with over 10,000 patients, our results are based on the by far largest population of human subjects for which NBSL distributions have been reported, which allows us to study the location specific distribution with an unprecedented level of detail. Apart from that, our study demonstrates a significant effect of NBSL on VF pattern deviation values.To minimize the potential effects of VF loss induced by glaucoma, we only selected visual fields with a normal or close to normal global VF sensitivity (mean deviation ≥−1 dB) and for which HFA parameters did not denote signs of glaucomatous vision loss.

In general, the distribution of blind spots we reported is asymmetric as shown in Fig. 2. In particular, it is skewed with a heavy tail in horizontal direction and bimodal in vertical direction. To our knowledge, we are the first to report on a bimodal distribution of NBSL in vertical direction. However, in previous studies a possible bimodality would not have been detectable due to their small samples sizes (30 and 104 eyes in the works by Safran *et al*. and Rohrschneider, respectively) compared to the 11,449 eyes used in our work.

The discrepancy of the average NBSL between our results and the results from previous studies^20, 21^ may suggest the blind spots distribution varies between populations, and variation of the peak location of blind spots can be larger in horizontal direction. Note that the two data sample of the two previous studies were normal healthy individuals, while we used a convenience sample from patients visiting glaucoma services which we suspect do not have functional loss bye virtue of the inclusion criteria we set. Thus the participants of our dataset are not typical healthy patients, which may also explain the discrepancy of the NBSL between our study and the previous two studies. In addition, the NBSL or optic disc location measured in the two previous studies are not based on HFA test. The HFA is a key device for clinical visual field testing which routinely provides NBSL results to aid clinicians with the interpretation of VF measurements, which also measures the blind spot location. We provide some basic descriptions of the HFA blind spot test in the Methods section which were obtained by close observation of the procedure. However, the precise details of this adaptive test are proprietary and not available to researchers. Therefore, comparisons with existing NBSL studies that did not apply the HFA need to be considered with care, as differences in the results could be in part due to different NBSL testing algorithms. Our results are close to an existing study which did not apply the HFA but used a well-described blind spot detection procedure instead^20^. Furthermore, there are close similarities even to the results of a study which determined the location of the optic nerve head relative to the fovea, i.e. a physiological correlate to NBSL^21^.

We observed a negative correlation between NBSL distance from fixation and spherical equivalent of refractive error. As the patient became more myopic, the distance between NBSL and fixation increased and vice versa. This is consistent with findings in literature that increased axial length of the eye was associated with myopia^22, 23^ and the optic disc-fovea distance was reported to be positively correlated to ocular axial length^24^. In addition, we compared the average PD difference of two groups which were divided based on NBSL distances. For the group with longer NBSL distance, average PD values decreased in the upper central and increased in the lower nasal VF region. Similarly, we also compared the average PD difference of two groups which were divided based on NBSL angles. For the group with more positive NBSL angles, average PD values decreased in lower nasal and increased in upper temporal VF areas.

The NBSL effects on PD reported in Fig. 4 might be confounded by possible fixation losses. To disentangle the impacts of fixation losses and of NBSL, we performed multivariate linear regressions from NBSL distance/angle and fixation loss rate to PD fields. Figure 5(a) and (c) show the respective NBSL distance/angle effects for those parts of variance which are not explained by fixation loss rate. The basic PD difference patterns closely resemble the respective patterns of Fig. 4, with even more significant locations.

Prior work has demonstrated that the position of the optic disc relative to the fovea has an impact on the RNFL trajectories, here we demonstrate that the NBSL relative to fixation (the functional equivalent of the position of the optic disc relative to the fovea) has a systematic effect on the spatial distribution of VF sensitivity. Accounting for the distance between optic disc and fovea is important for inferring VF sensitivity based on optic nerve structural characteristics in health and disease, individual NBSL information which implicitly represents the distance between optic disc and fovea should be taken into consideration for assessing VF loss.

## Methods

The visual field measurements used for this study were obtained by the Glaucoma Research Network, a consortium including five large clinical glaucoma centers in the US from: Massachusetts Eye and Ear, Wilmer Eye Institute, New York Eye and Ear Infirmary, Bascom Palmer Eye Institute and Wills Eye Hospital. This retrospective study was approved by the institutional review boards of those five aforementioned institutions.

### Subjects and Data

From an initial dataset of Swedish interactive thresholding algorithm (SITA) Standard 24–2 VFs measured with the HFA, the most recent reliable VFs were selected from each eye. The reliability criteria for VF selection were fixation loss ≤33%, false negative rates ≤20% and false positive rates ≤20%, which are used in clinical practice at Massachusetts Eye and Ear. It has been reported that the HFA underestimates the false positive rates of the patients, particularly among normal patients^25^. We used a more conservative threshold (20%) for false positive rates than the HFA default (33%), which was also used in a number of previous studies^26–30^. For data analysis, left eyes were transposed to right eye format. To minimize possible effects of potentially manifest glaucomatous VF loss, only VFs that are clinically unaffected with mean deviations ≥−1 dB, pattern standard deviations (not pattern deviation) that are not flagged as abnormal and glaucoma hemifield tests within normal limits were included. If both eyes of a patient met our selection criteria, one eye was randomly chosen.

We extracted the pattern deviation (PD) plot values from each of the 54 locations tested as part of the 24–2 pattern. PD values represent the deviations in decibels from the age corrected normal values of a healthy population at each tested point in the visual field adjusted for the individual overall sensitivity by subtracting the 85th percentile^31^. The HFA expects a default blind spot location at (15°, −1°) of visual angle and omits the two locations closest to this point for calculating PD values, therefore, 52 locations were considered in this study.

### Blind Spot Testing

At the beginning of each perimetric test of the HFA 24–2 protocol, blind spot localization is routinely performed and the result of which is denoted on the VF printout by a triangle^19^. The center of the tested blind spot, which defines NBSL in our work, can be exported as a numerical value from the machine. The spatial resolution of the reported blind spot centers is one degree of visual angle (see Fig. 1).

The HFA blind spot test is a proprietary adaptive method the details of which were not available from the manufacturer upon request (personal communication with Carl Zeiss Meditec). The following details about the test are therefore derived from our own observations of the testing procedure. The HFA NBSL test implements the same response procedure as the subsequent VF test, i.e. the subject fixates at a central fixation mark and is instructed to press a response button whenever a visual stimulus is visible. Visual stimuli are white Goldmann-size III dots presented at the HFA maximal luminous intensity (0 dB). The adaptive test always starts at location (15°, −1°) for right eyes and (−15°, −1°)for left eyes. The dots are presented with fixed inter-stimulus intervals on a regular grid with one degree distance between the grid locations. The precise order of the presentation is undocumented but depends on the subject’s response behavior.

When the test fails, for instance because the subject presses the response button for every single stimulus presented, which is a realistic scenario if the second eye is not sufficiently occluded, the HFA raises a warning to the operator. However, this warning is not reported on the VF test printout. Instead, for failed NBSL tests, the HFA returns the “default” location of (15°, −1°) for right and (−15°, −1°) for left eyes. Blind spot testing can be switched off by the HFA operator, for instance to save time. In this case, the HFA reports the “default” location as well. If a location of (15°, −1°) for right eyes or (−15°, −1°) for left eyes is reported by the HFA, it is indistinguishable whether this location is the true testing result or rather indicates a failed or omitted test. Therefore, we chose to exclude all cases with this test result.

For subsequent data analyses, in particular for the Gaussian Process Regression used to model the NBSL distribution, we additionally excluded blind spot locations with frequency counts less than 5, which tended to occur at locations with large eccentricity and which are likely to be outliers due to artifacts.

### Statistical Modelling and Analysis

All statistical modelling was performed by R platform and Octave software^32, 33^.

#### Gaussian Process Regression

To estimate a continuous distribution of NBSL from discrete NBSL measurements at grid locations, Gaussian process regression was used to compute the continuous distribution of the frequency of eyes at each discrete NBSL^34^. Typically Gaussian process regression can give the best linear unbiased prediction for unknown spatial locations. Herein we briefly describe the concept of Gaussian process regression. Details can be found in the literature^35^.

For a Gaussian process, each entry of the *n* observations **y** = {*y*_1_, …, *y*_*n*_} in spatial domain ${\rm{\Omega }}(\overrightarrow{x})$ is associated with a normally distributed random variable, and a finite collection of those random variables has a multivariate normal distribution. Thus, the expected value of the unobserved variable y_*_ at spatial location can be interpolated by maximizing the probability of y_*_ conditioning on known *n* observations **y** assuming [**y**, y_*_] to be a multivariate normal distribution. Gaussian process regression relies on a covariance function that represents the spatial relationship between the samples. Here, we apply a frequently chosen covariance function $k\,({\overrightarrow{x}}_{i},{\overrightarrow{x}}_{j})$ defined as follows:

1 $$k\,({\overrightarrow{x}}_{i},{\overrightarrow{x}}_{j})={\sigma }_{f}^{2}exp\,[\frac{-{\Vert {\overrightarrow{x}}_{i}-{\overrightarrow{x}}_{j}\Vert }^{2}}{{\overrightarrow{\gamma }}^{2}}]$$

where *σ*_*f*_ defines the maximum allowable covariance, $\overrightarrow{\gamma }$ is the length scale of the kernel and ||.|| is the *l*_2_ norm^35–37^. *sigma*_*f*_ and $\overrightarrow{\gamma }$ are chosen by optimizing the posterior probability of *σ*_*f*_ and $\overrightarrow{\gamma }$ based on the observational data.

#### Group Comparison of PD Fields

To illustrate the variation of PD distribution with respect to the NBSL distance relative to fixation, we divided the patients into two groups which had NBSL distances longer or equal than the median of all NBSL distances and NBSL distances shorter than the median of all NBSL distances. The mean PD values at each VF measurement location of the two groups were compared by the differences. Similarly, the patients were divided into two groups which had NBSL to fixation angles larger or equal than the median of the all NBSL angles and NBSL to fixation angles smaller than the median of the all NBSL angles. The difference of the PD values of the two groups was compared. Multiple comparisons correction for p-values were adjusted by false discovery rate^38^.

#### Multivariate Linear Regression of PD Fields

To elucidate whether the spatial pattern of the association between NBSL distance/angle and PD fields are independent of the potential confounding factor “fixation loss”, multivariate linear regression is applied to associate PD fields with NBSL distance/angle and fixation loss rate. The regression coefficient signs for NBSL distance/angle and fixation loss rate are shown for all 52 VF testing locations. Multiple comparisons correction for p-values were adjusted for the regression coefficients by false discovery rate as well^38^.

### Data availability statement

The datasets generated during and/or analysed during the current study are available from the corresponding author (Dr. Elze) on reasonable request.
